# Use of Population Genetics to Assess the Ecology, Evolution, and Population Structure of *Coccidioides*

**DOI:** 10.3201/eid2206.151565

**Published:** 2016-06

**Authors:** Marcus M. Teixeira, Bridget M. Barker

**Affiliations:** Translational Genomics Research Institute, Flagstaff, Arizona, USA (M.M. Teixeira, B.M. Barker);; Northern Arizona University, Flagstaff (B.M. Barker); University of Arizona, Tucson, Arizona, USA (B.M. Barker)

**Keywords:** Valley fever, coccidioidomycosis, microsatellites, Coccidioides immitis, Coccidioides posadasii, Arizona, fungi, fungus, population genetics, population structure, United States

## Abstract

During the past 20 years, a general picture of the genetic diversity and population structure of *Coccidioides*, the causal agent of coccidioidomycosis (Valley fever), has emerged. The genus consists of 2 genetically diverse species, *C. immitis* and *C. posadasii*, each of which contains 1 or more distinct populations with limited gene flow. Genotypic data indicate that *C. immitis* is divided into 2 subpopulations (central and southern California populations) and *C. posadasii* is divided into 3 subpopulations (Arizona, Mexico, and Texas/South America populations). However, admixture within and among these populations and the current paucity of environmental isolates limit our understanding of the population genetics of *Coccidioides*. We assessed population structure of *Coccidioides* in Arizona by analyzing 495 clinical and environmental isolates. Our findings confirm the population structure as previously described and indicate a finer scale population structure in Arizona. Environmental isolates appear to have higher genetic diversity than isolates from human patients.

## Abstract 2

Although *Coccidioides* genotypes are highly genetically variable, they cluster into discrete populations, which has implications for human infections.

*Coccidioides immitis* and *C. posadasii* are the only 2 species recognized within the genus *Coccidioides* (*1*). These fungi are endemic to arid or semi-arid regions of the Americas. Both species cause the disease coccidioidomycosis (Valley fever), which is contracted by dogs, humans, and other mammals living in or visiting *Coccidioides*-endemic areas (*2*,*3*). Infection is acquired through inhalation of air-dispersed arthroconidia (asexual single-cell fungal propagules). When a mammalian host inhales these conidia, a switch from polar to isotropic growth is initiated, resulting in the development of a specialized infectious structure called a spherule (*4*). Within 4 to 5 days, the mature spherules disrupt, releasing potentially hundreds of endospores, each of which are capable of developing into a new spherule (*5*). This cycle continues until the host’s immune system represses fungal propagation or the fungus goes quiescent (*4*). If infection is not controlled, it can disseminate to other organs and tissues and is capable of crossing the blood–brain barrier and causing meningitis, which is fatal if untreated (*6*). Approximately 40% of infections are symptomatic (*4*).

The geographic distribution of *C. immitis* was thought to be restricted to central and southern California (*7*). However, the range extends south into Baja California and east into Arizona, and recent work shows this species was also found in eastern Washington (*8*,*9*), at Dinosaur National Monument in Utah (*10*), and in a patient in Colombia with no travel history (*11*). The species *C. posadasii* is present in Arizona, with its range extending into Utah, Texas, and Mexico and dispersed populations in Central and South America (*12*–*15*). *C. immitis* and *C. posadasii* probably co-occur in nature, given that both species have been isolated from patients in San Diego and Mexico and hybrid strains have been identified (*1*,*16*). Environmental sampling and recovery of isolates would be more helpful in confirming this hypothesis than using isolates derived from patients.

One approach to assessing genetic diversity in fungal populations is to develop microsatellite markers (*17*,*18*). Microsatellites are short (1–6 bp) tandem repeats, which are found throughout eukaryotic genomes and are thought to be evolving under neutrality in fungi (*19*). These markers have been useful in population genetics studies that compare genotypes among closely related fungal species or populations (*17*,*20*–*23*). Here we focus on the genotyping of *Coccidioides* strains from various origins by combining multiple studies in a meta-analysis and by using population genetics to clarify the causative agents of coccidioidomycosis.

Because coccidioidomycosis is increasing and disease severity is highly variable, defining genotypic distribution is important for monitoring outbreaks and determining whether increased pathogenicity is an emerging trait (*24*). Previous analysis showed that a single clone did not cause the rise in infection rates in Arizona; rather, each isolate recovered from a patient was unique (*25*). Thus, the question remains: why is coccidioidomycosis on the rise? It has been hypothesized that climate change, changes in human susceptibility, changes in reporting, or a result of the interaction of these factors, overlaid with high genetic variation and the possibility that *Coccidioides* can colonize new hosts and new environments, are some of the factors responsible (*3*,*26*,*27*). We aimed to answer 4 main questions: 1) if the subpopulation structure previously proposed has support when a larger dataset is analyzed by using multiple methods; 2) if there is evidence for population structure within Arizona; 3) if environmental isolates from Arizona are distinct from Arizona human host isolates; and 4) if patient data confound population structure because of incorrect identification of the point source of infection.

## Methods

### Strains

In total, we compiled data from 66 soil-derived isolates retrieved by mouse passage in Tucson, Arizona (*28*); 141 isolates from Arizona patients with Valley fever (*25*); 106 *C. posadasii* and 62 *C. immitis* isolates from a broad geographic range (*1*); and 266 clinical *C. posadasii* isolates (human and veterinary) newly analyzed for this study (Technical Appendix 1 Table). Of these 641 isolates, 22 were removed from final analysis for failure to amplify >2 of the 9 loci. 

### DNA Extraction

To extract DNA, we placed ≈0.2 g of mycelia in a 2-mL screw-cap tube containing 0.5-mm–diameter sterile glass beads (BioSpec, Bartlesville, OK, USA) and 1 ml of lysis buffer (50 mmol/L Tris-HCl [pH 7.5], 100 mmol/L EDTA [pH 8.0], 100 mmol/L NaCl, 0.5% sodium dodecyl sulfate, and 100 mmol/L β-mercaptoethanol) and subjected it to mechanical disruption by vortexing on a flat 12-tube holder (MoBio, Carlsbad, CA, USA) at 3,700 rpm for 10 minutes. Samples were incubated at 65°C for 60 minutes and centrifuged at 8,000 rpm for 5 minutes. We extracted nucleic acids from the supernatant with buffered phenol:chloroform:isoamyl alcohol pH 8.0 (25:24:1) and again with cholorform:isoamyl (24:1) and precipitated from the aqueous layer with 0.6 volumes of isopropyl alcohol. We washed the pellets twice with ethanol and resuspended them in 150 µL of double-distilled H_2_O. DNA concentration was determined on NanoDrop 1000 spectrophotometer (Thermo Scientific, Wilmington, DE, USA) and was diluted to 20 ng/µL.

### Multilocus Microsatellite Typing Markers and PCR

To genotype isolates, we used 9 microsatellite primers developed for phylogenetic analysis and tested for concordance in *Coccidioides* (*17*,*18*,*25*). All microsatellite fragments were first denatured for 2 min at 96°C, followed by 30 amplification cycles (30 s at 94°C, 30 s at 55°C, and 1 min at 72°C) and 1 extension cycle of 5 min at 72°C with 2.5× Hotmaster mix (Eppendorf, New York, NY, USA). One primer from each set was end-labeled with a fluorescent tag (either NED dye [ABI, Shirley, NY, USA] or FAM or HEX [Eurogentec, Seraing, Belgium). Primer concentrations were 200 nmol/L each per reaction, and 100 ng of DNA was used for each reaction.

### Fragment Analysis

We grouped microsatellite fragments from each isolate into 3 sets of 3 fragments and labeled 1 primer set in each grouping with HEX, FAM, or NED. Pooled PCR products were separated on an ABI 3730 DNA Analyzer (Applied Biosystems, Foster City, CA, USA) at the University of Arizona Genomic Analysis and Technology Core sequencing facility, using a ROX-labeled ladder (Invitrogen, Carlsbad, CA, USA) for sizing. Chromatographs were read in Genotyper (ABI, Shirley, NY, USA), and a single peak was scored (*Coccidioides* is haploid). No evidence of multiple peaks was detected. Microsatellites were amplified and analyzed at least twice to verify their size. To compare our isolates to those described in published data, we analyzed the microsatellite sizes from a subset of previously analyzed isolates on our ABI 3730 system (Technical Appendix 1 Table, duplicates tab). Calibration was necessary to compare the published microsatellite sizes to our data (Technical Appendix 1 Table, correction tab).

### Population Analyses

We tabulate data from the Genotyper program maintained them in a spreadsheet (Technical Appendix 1 Table). Files were checked for duplicates and clone–correction checked using GenAlEx 6.501 (*29*). We found identical isolates from multiple isolations from the same patient and from isolates collected from the same soil site. Any samples that were missing >3 loci were eliminated from the final dataset. Locations were incorporated into a nexus file containing 619 isolates. We assigned locations based on the isolation/hospital origin as follows: Phoenix, Yuma, and Tucson (Arizona); San Diego and San Joaquin Valley (California); Texas; Mexico; and South America (Brazil, Argentina, and Venezuela).

We analyzed microsatellite matrices by using STRUCTURE 2.3.4 (Pritchard Laboratory, Stanford University, Stanford, CA, USA) to determine population structure within *Coccidioides* (*30*). The running length of burn-in period was 100,000 repetitions with 1,000,000 Markov chain Monte Carlo repetitions. Default settings in STRUCTURE 2.3.4 were as follows: the admixture model was used to infer α along with the previous sampling location information model (LOCPRIOR) (*30*). We used CLUMPP, a cluster matching and permutation program (https://web.stanford.edu/group/rosenberglab/clumpp.html), to define populations within the STRUCTURE algorithm. K is the number of significant populations in each main group. Allele frequencies were assumed to be correlated among populations, assuming that there are different Fst values for different subpopulations, the previous mean of Fst for populations is 0.01, and λ is constant at 1.0. Ten runs for each *k* from 1 to 10 were performed, and results were analyzed using Evanno’s method implemented in StructureHARVESTER (*31*). We generated a consensual STRUCTURE plot from the admixture values using the Clustering Markov Packager Across K (CLUMPAK) (http://www.clumpak.tau.ac.il) and built final plots with STRUCTURE PLOT (*32*,*33*).

We also inferred *Coccidioides* population splits and mixtures trees using a statistical model related to common ancestors through a graph of ancestral populations via TreeMix software (Pritchard Laboratory) (*34*). In brief, we inferred a population tree on the basis of microsatellite data for each of the identified populations in STRUCTURE (Technical Appendix 2 Table). Migration events were placed on admixed edges, which are correlated with the degree of ancestry for each population and represents unidirectional gene flow between populations. Horizontal branch lengths are proportional to the accumulated genetic drift (drift parameter) from each population that was placed in a given branch. The drift parameter measures the variance in allele frequency that changes along each population of the tree. We also analyzed the same data were by using Nei’s unbiased genetic distance estimate (Table 1), to complete a principal coordinate analysis (PCoA) (Table 2) in GENALEX 6.501 (http://www.biology-assets.anu.edu.au/GenAlEx/Welcome.html) (*29*). We documented allele frequencies, private alleles, and haploid diversity calculations (Table 3) for Arizona samples (Technical Appendix 2 Table).

**Table 1:** Pairwise population matrix of Nei’s unbiased genetic distance for principal coordinates analysis of *Coccidioides* populations, Arizona, USA*

**Population**	**PHOENIX**	**AZSOIL**	**TUCSON**	**SJV**	**SDMX**	**MEXICO**	**TXSA**
**PHOENIX**	0.000						
**AZSOIL**	0.128	0.000					
**TUCSON**	0.158	0.277	0.000				
**SJV**	2.582	2.571	1.675	0.000			
**SDMX**	2.519	2.570	1.737	0.143	0.000		
**MEXICO**	0.354	0.324	0.477	1.480	1.546	0.000	
**TXSA**	0.602	0.638	0.526	1.580	1.734	0.373	0.000

*The larger the genetic distance value, the greater the genetic difference between populations. PHOENIX represents primarily *Coccidioides posadasii* human clinical isolates from Yuma and Phoenix, Arizona. AZSOIL represents primarily environmental and veterinary clinical *C. posadasii* isolates from Arizona. TUCSON represents primarily human clinical *C. posadasii* isolates from Tucson, Arizona. SJV represents primarily *C. immitis* human clinical isolates from Bakersfield, California. SDMX represents primarily *C. immitis* human clinical isolates from San Diego, California, and Mexico. MEXICO represents primarily human clinical *C. posadasii* isolates from Mexico. TXSA represents primarily human clinical *C. posadasii* isolates from Texas, Brazil, Argentina, and Venezuela.

**Table 2:** Principal coordinates analysis results indicating percentage of variation among *Coccidioides* populations, Arizona, USA

**Value**	**Axis**		
	1	2	3
**% Variation**	93.92	3.95	1.44
**Total eigenvalue**	1.202	0.051	0.018
PHOENIX	0.491	−0.061	0.009
AZSOIL	0.498	−0.018	0.009
TUCSON	0.213	−0.012	−0.103
SJV	−0.552	0.089	−0.023
SDMX	−0.552	−0.098	0.022
MEXICO	0.162	0.048	0.081
TXSA	0.179	0.163	−0.001

*PHOENIX represents primarily *Coccidioides* posadasii human clinical isolates from Yuma and Phoenix, Arizona. AZSOIL represents primarily environmental and veterinary clinical *C. posadasii* isolates from Arizona. TUCSON represents primarily human clinical *C. posadasii* isolates from Tucson, Arizona. SJV represents primarily *C. immitis* human clinical isolates from Bakersfield, California. SDMX represents primarily *C. immitis* human clinical isolates from San Diego, California, and Mexico. MEXICO represents primarily human clinical *C. posadasii* isolates from Mexico. TXSA represents primarily human clinical *C. posadasii* isolates from Texas, Brazil, Argentina, and Venezuela. Axis 1 explains 93.92% of the genetic variation among the populations, which is due mainly to separation of *C. immitis* (SJV and SDMX) from *C. posadasii.* Axis 2 suggests gene flow between SJV, MEXICO, and TXSA. Axis 3 indicates that the TUCSON population contains unique genetic signatures.

**Table 3:** Summary of diversity indices for the *Coccidioides posadasii* population, Arizona, USA*

**Source of isolate**	**No.**	**Different alleles**	**Effective alleles**	**Shannon’s informative index**	**Diversity**	**Unbiased diversity**	**Private alleles**
**Tucson clinic**							
Mean	251.444	9.444	3.139	1.288	0.581	0.584	20
SE	3.158	1.676	0.533	0.220	0.085	0.085	
**Yuma clinic**							
Mean	9.000	3.333	2.568	0.905	0.480	0.540	0
SE	0.000	0.577	0.473	0.204	0.101	0.114	
**Phoenix clinic**							
Mean	128.333	6.889	2.952	1.169	0.554	0.558	5
SE	1.546	1.296	0.560	0.207	0.083	0.084	
**Soil**							
Mean	64.778	6.667	3.155	1.257	0.598	0.607	4
SE	1.103	1.118	0.529	0.192	0.077	0.078	
**Veterinary**							
Mean	13.556	4.222	2.748	1.044	0.528	0.571	0
SE	0.338	0.703	0.437	0.199	0.091	0.099	

*Tucson clinic isolates are all human clinical isolates from Tucson, Arizona. Yuma clinic isolates (previously included in the PHOENIX population) are all human clinical isolates from Yuma, Arizona. Phoenix clinic isolates are all human clinical isolates from Maricopa County, Arizona. Soil isolates are the 66 environmental isolates from Tucson, Arizona, previously grouped in AZSOIL. Veterinary isolates are from various host animals, previously grouped in the AZSOIL population.

## Results

### Combining Data from Multiple Sources

**Figure 1:**
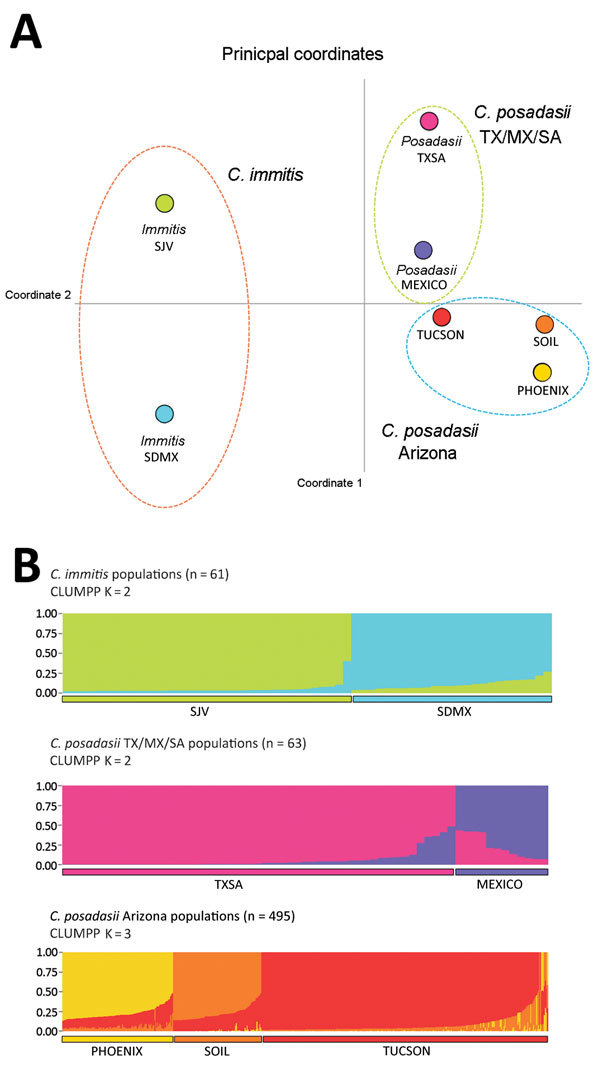
Results of principal coordinate analysis and STRUCTURE analyses of *Coccidioides spp.* populations. A) Principal coordinate analysis using Nei’s unbiased genetic distance estimates supports 3 main groupings: *C. immitis, C. posadasii* TX/SA/MX, and *C. posadasii* Arizona (see also Technical Appendix 3 Figure 2). The greatest separation occurs between species and is reflected in principal coordinate 1 (93.92% of variance). Color-coding for populations: lime green, San Joaquin Valley (SJV); aqua, San Diego/Mexico (SDMX); pink, Texas/South America (TXSA); purple, Mexico (MEXICO); red, Tucson (TUCSON); yellow, Phoenix/Yuma (PHOENIX); orange, soil (AZSOIL). B) STRUCTURE analysis. Microsatellite matrices were analyzed with STRUCTURE 2.3.4 to determine population structure within *Coccidioides* populations (*30*). The running length of burn-in period was 100,000 repetitions with 1 million Markov chain Monte Carlo repetitions. Default settings in STRUCTURE 2.3.4 were as follows: the admixture model was used to infer α along with the previous sampling location information model (LOCPRIOR) (*30*). We used CLUMPP, a cluster matching and permutation program (https://web.stanford.edu/group/rosenberglab/clumpp.html), to define populations within the STRUCTURE algorithm. K is the number of significant populations in each main group. A consensual STRUCTURE plot was generated from the admixture values by using the Clustering Markov Packager Across K (CLUMPAK) server, and final plots were built with STRUCTURE PLOT (*32*,*33*).

We documented microsatellite frequencies (Technical Appendix 3 Figure 1; Technical Appendix 1 Table). Three loci (GAC2, 621.1, and ACJ) had low diversity in *C. posadasii*, and these same loci were variable in *C. immitis*. Three loci showed the opposite pattern (K01, K03, and K07) and had low diversity for *C. immitis* and are variable in *C. posadasii*. Three loci (K09, GA1, and GA37) were diverse for both species. These results were similar to those of earlier reports (*1*). We merged datasets were merged for analysis (Technical Appendix 1 Table) and analyzed isolates from both published datasets (Technical Appendix 1, duplicates tab). Manual corrections of 1 or 2 bp were needed because of slight variations among machines and ladders (Technical Appendix 1, corrections tab).

### Population Structure of *Coccidioides* Subspecies

**Figure 2:**
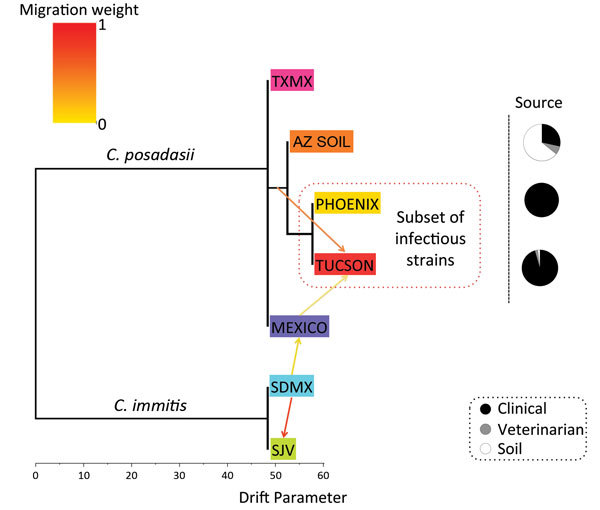
Population tree of *Coccidioides* subspecies population splits and mixtures. Maximum-likelihood population tree and presence of gene flow between diverged *Coccidioides* populations were inferred by using TreeMix software and microsatellites data (*34*). Direction of arrow indicates migration or gene flow based on admixture models; migration weights are shaded according their importance, supporting gene flow from a soil-derived population (AZSOIL) recovered from animal passage to a clinical-associated population (TUCSON). Color-coding for populations: lime green, San Joaquin Valley (SJV); aqua, San Diego/Mexico (SDMX); pink, Texas/South America (TXSA); purple, Mexico (MEXICO); red, Tucson (TUCSON); yellow, Phoenix/Yuma (PHOENIX); orange, soil (AZSOIL). The drift parameter, represented by horizontal scale, measures the variance in allele frequency change along each branch of the tree. The actual source of each evaluated isolate (clinical, veterinary, or soil) is represented proportionally in the pie chart.

STRUCTURE analysis based on 619 isolates revealed 3 *Coccidioides* populations for *C. immitis* (n = 61), *C. posadasii* Mexico/Texas/South America (n = 63), and *C. posadasii* Arizona (n = 495) (Technical Appendix 3 Figure 2). We detected low gene flow between the 3 major populations as observed by unique bar plots for each of these populations and observed gene flow between *C. posadasii* Mexico/Texas/South America and *C. posadasii* Arizona and between *C. immitis* and *C. posadasii* Mexico/Texas/South America (Technical Appendix 3 Figure 2). The population tree displays the 3 main populations and population assignments for each isolate along the bar plots (Technical Appendix 3 Figure 2). PCoA analysis using Nei’s unbiased genetic distance estimates revealed 3 main groupings when considering all data (Figure 1, panel A). Principal component (PC) 1 explains 93.92% of the variation, mainly attributable to variation between the species (Eigen value 1.202). PC2 explains 3.95% of the variation, reflecting the subpopulation structure in both species (Eigen value 0.051). PC3 explains 1.44% of variation and further separates Mexico from Arizona (Table 2).

### Population Structure within *C. immitis* Population

**Figure 3:**
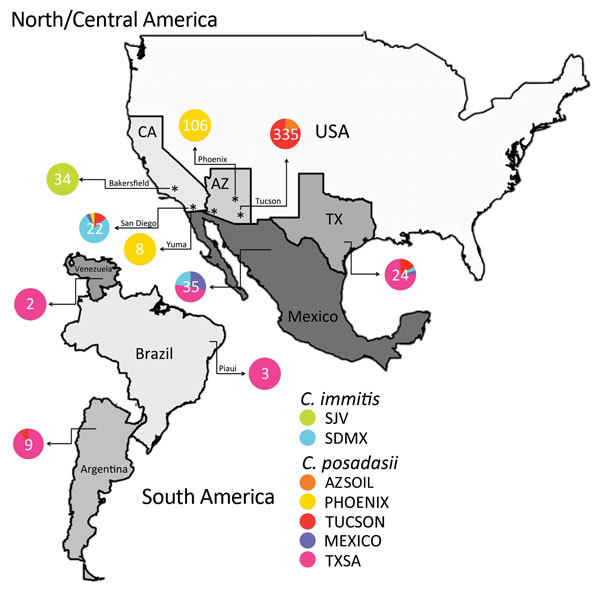
*Coccidioides* subspecies distribution for North, Central, and South America. The frequency of assignment for each *Coccidioides* population was plotted in a pie chart for each location, and numbers of isolates from each location are displayed. For example, patients from Mexico were infected with isolates from Texas, San Diego, and Mexico populations, as determined by analysis with STRUCTURE. Because each of the patients’ location is the hospital, no fine-scale population is assessed.

Results of PCoA analysis strongly indicated population structure within *C. immitis*, separating San Joaquin Valley (SJV) from San Diego and Mexico (SDMX) isolates (Figure 1, panel A). STRUCTURE analysis also indicates a strong population subdivision within *C. immitis* (Figure 1, panel B). According to the optimal number of clusters determined by using StructureHARVESTER, the SJV and SDMX isolates are clustered into 2 different populations (*k* = 2) (Figure 1, panel B; Technical Appendix 3 Figure 3). Bar plots show that limited gene flow was observed between these subpopulations; however, the bar plots also indicated that the *C. immitis* isolates 17TX, 4SD, 8SD, 4M3, and 8M3 share alleles from both populations (Technical Appendix 3 Figure 3). The population tree indicates that the *C. immitis* SJV population has a migration event from the *C. immitis* SDMX population (Figure 2). The isolate population distribution frequency for *C. immitis* reveals differences between SDMX and SJV populations (Figure 3).

### Population Structure within *C. posadasii* Mexico/Texas/South America Population

For *C. posadasii* Mexico/Texas/South America population, we detected 2 optimal clusters (Technical Appendix 3 Figure 3), 1 including Texas/South America isolates and 1 constituting isolates from Mexico (Figure 1). The Mexico isolates display a high level of hybridization between 2 different populations as well as within *C. immitis* (Technical Appendix 3 Figures 1, 3). The migration event from *C. immitis* SDMX to *C. posadasii* Mexico and the more basal *C. immitis* to *C. posadasii* Tucson migration event implicate the Sonoran desert as a convergent source of multiple *Coccidioides* genotypes and possible center of origin of the genus (Figures 2, 3).

### Population Structure within *C. posadasii* arizona Population

Population structure analysis of 495 separate fungal isolates suggests at least 3 different *C. posadasii* subpopulations in Arizona, in agreement with PCoA data (Figure 1). Clinical samples from Yuma and Phoenix (designated PHOENIX) and Tucson patients (designated TUCSON) fall in 2 different populations according to STRUCTURE (Figure 1, panel B). All environmental samples and some veterinary/clinical samples from Tucson, Phoenix, and Yuma regions constitute a third population (designated AZSOIL) apart from the TUCSON and PHOENIX populations (Figure 1; Technical Appendix 3 Figure 3). We detected high level of admixture in the Arizona population, suggesting gene flow between 3 populations. However, the presence of private alleles for different loci within each of the 3 Arizona populations supports genetic isolation (Technical Appendix 2 Table, AZ_PAL tab). Structure plots of AZSOIL, PHOENIX, and TUCSON populations contain isolates with genotypes from all 3 populations (Technical Appendix 3 Figure 3). AZSOIL and TUCSON populations arose from the same geographic origin (Figure 3). The population tree (Figure 2) supports a migration event from AZSOIL to TUCSON. The AZSOIL is placed nearer to the ancestral branch for Arizona subpopulations. In addition, a low number of clinical isolates clustered with AZSOIL, leading us to consider variable pathogenicity or host specificity (Figure 2). We propose that a mammalian host or its close microenvironment (e.g., mammal burrows) could contribute to increased fitness of a virulent phenotype. Thus, the environmental reservoir could play a role in the emergence of pathogenic strains.

### Clinical Isolates

Data obtained from genotyping human patient isolates might lead to incorrect estimates of population structure. Two *C. immitis* were found in patients in Phoenix hospitals, and both patients had confirmed travel to California; however, we analyzed only 1 because the other did not meet our cutoff criteria (*25*). A Texas patient isolate was determined to be *C. immitis* (*1*). Patients from China, Switzerland, and Colorado (1 patient from each) and 7 California patients were infected with *C. posadasii* (*1*). One of the widely used laboratory *C. posadasii* strains (Silveira) was isolated from a patient with coccidioidomycosis diagnosed in California. In northern Mexico (including Baja California) and southern Mexico (Michoacán state), many strains are genotyped as *C. immitis* but have evidence of hybridization with *C. posadasii* and signatures of introgression (*16*). Less is known about the prevalence of introgression found in the *C. posadasii* Mexico population. For Arizona isolates newly analyzed for this study, no *C. immitis* were identified (Technical Appendix 1 Table).

## Discussion

Multiple methods and previous reports show that there are 2 species within *Coccidioides* defined as *C. posadasii* and *C. immitis* (*1*,*7*). Within species, *C. posadasii* contains the 2 main populations of Texas/South America/Mexico and Arizona, and within *C. immitis*, 2 populations are suggested, SJV and SDMX, supported by our data and previous reports (Figure 1) (*7*). Gene flow between *C. immitis* populations is not abolished, as exemplified by the admixture isolates 17TX and 22SD (Technical Appendix 3 Figure 3). STRUCTURE analysis suggests that *C. posadasii* Arizona and Texas/Mexico/South America populations are highly differentiated, with few isolates sharing genotypes among them (Technical Appendix 3 Figure 3). Additionally, divergence between Mexico and South America/Texas is evident, such that they are evolving independently (Figure 1).

Within the Arizona population, we observed 3 clusters: PHOENIX, TUCSON, and AZSOIL (Figure 1). PHOENIX consistently groups separately from TUCSON and AZSOIL, which might reflect differences in ecology between Arizona upland (Tucson) and the Lower Colorado Valley (Phoenix and Yuma) or variation in pathogenicity among hosts. Variation in mean soil temperature, precipitation, natural hosts, and vegetation could exert differential selection pressure on the fungus in the environment (*35**,**36*). In addition, according to the population tree, the AZSOIL subpopulation appears to be basal within Arizona. The migration event from AZSOIL to TUCSON might reflect selection of more pathogenic genotypes because only ≈40% of infections are symptomatic (*4*), and even fewer of these would result in severe disease where the isolate would be collected from the patient (Figure 2). This leads us to propose that the AZSOIL subpopulation reflects greater diversity than the TUCSON and PHOENIX subpopulations and that this greater diversity might be driven by selection of certain pathogenic strains in humans.

Moreover, our soil sampling reflects diversity at only 7 locations in and around Tucson, and all samples were collected with a single year, whereas the patient isolates from Tucson were collected over a period of 30 years. These soil isolates were obtained using a highly sensitive murine model of coccidioidomycosis. Not all mice had evidence of illness, and infection was only realized upon necropsy. Thus, we might have selected for infectious strains, but we believe we captured diversity in pathogenesis. This assumption would suggest that we have underestimated diversity in the environment. Diversity at some soil locations was high (i.e., multiple genotypes were recovered), whereas other sites were clonal, or we only recovered a single colony. Thus, it was surprising to find higher unbiased genetic diversity in AZSOIL (0.607, ±0.078 SE) than in TUCSON (0.584, ±0.085 SE) (Table 3). Patient isolates can provide information on a coarse level, but finer-scale mapping of geographic and population boundaries will require environmental sampling and analysis of genotypes. Our data suggest that environmental isolates reflect a broad diversity of genotypes and only a subset may be capable of causing severe disease in humans. A primary concern with our analysis is the precise location of the isolate origin. Few environmental isolates of *Coccidioides* exist, and methods to obtain them for genotypic analysis are currently inadequate (*9*,*10*,*28*,*36*).

Admixtures were found in the Arizona population, and gene flow was observed between the three defined subpopulations (Technical Appendix 3 Figure 3); however, the presence of private alleles within each of those subpopulations and high genetic distance supports genetic isolation (Tables 1–3). The same was observed for the Mexico isolates nested in the Texas/Mexico/South America population. Additionally, the presence of private alleles and high diversity within *C. immitis* suggests that our results are not affected by oversampling in the Arizona subpopulation (Table 3). Because a sexual life cycle has not been observed, questions related to frequency, timing, and directionality of genetic exchange remain to be explored experimentally. Additional multilocus microsatellite types might be needed to support populations or could be resolved by using whole genome sequence comparison. Our data support previous work identifying the same main populations (*1*,*7*) and can be further tested with additional single nucleotide polymorphisms identified using whole genome sequence comparison.

Questions remain about the population biology of *Coccidioides*. The spatial and temporal distribution of individual genotypes, the amount of spatial overlap between the 2 species, and population boundaries within each species are still unclear (*9*,*10*,*16*). Overlap between species is likely, because of the identification of both *C. immitis* and *C. posadasii* recovered among patients in San Diego and northern Mexico and the observation of hybridization and introgression (*1*,*7*,*16*). This work shows that analyzing a large number of patient isolates and assigning regional population information reveals the potential for population structure within Arizona, at a much finer scale than previously thought (Figure 1). Thus, genetic differences and population subdivision among isolates and populations are likely greater than has been shown to date. The question of which population is basal to the *C. posadasii* lineage remains unanswered, and greater efforts to explore genotypic variation in Texas, Mexico, and Central and South America are needed.

Understanding the ecology of *Coccidioides* has been a longstanding goal (*3*,*4*). We used multiple methods to understand population genetics and determine population structure (*29*,*30*). However, environmental isolates must be more deeply explored by using direct fungal isolation (not passaging in mice) or high-coverage metagenomic sequencing, so that a specific location can be assigned to each isolate and potential for greater genetic diversity in the environment could be specifically tested. Surveying human patient isolates will continue to be valuable to track new outbreaks, such as the current coccidioidomycosis cases in Washington State (*9*).

Investigating the ecology and distribution of genotypes within and among populations of a pathogen is important for monitoring outbreaks, determining variance in virulence, and predicting disease progression (*37*). Correlating disease severity with pathogen genotype by using genome-wide association studies might assist in identifying genetic-based differences in virulence (*38*). Monitoring disease progression and response to antifungal therapy in animal models of coccidioidomycosis with more than a few well-characterized laboratory strains might provide information that could assist with better treatment options (*39*). Finally, a better understanding of ecologic and environmental factors that influence the growth and reproduction of the organism will assist in predicting and preventing exposure to the pathogen (*28*).

Technical Appendix 1 Isolate details, calibration information for microsatellite data, and source files for STRUCTURE, TreeMix, and GenAlEx.

Technical Appendix 2 Processed GenAlEx data files.

Technical Appendix 3 Microsatellite allele frequencies and STRUCTURE plots displaying the 3 main populations of *Coccidioides* and 7 subpopulations.
