# Supplementary material for: Use of Population Genetics to Assess the Ecology, Evolution, and Population Structure of Coccidioides
Source: Emerg Infect Dis. 2016 Jun;22(6):1022–30. doi: 10.3201/eid2206.151565 (PMC4880095; doi:10.3201/eid2206.151565)
Supplement: Technical Appendix 3 — Microsatellite allele frequencies and STRUCTURE plots displaying the 3 main populations of Coccidioides and 7 subpopulations. [file 15-1565-Techapp-s3.pdf]

Article DOI: http://dx.doi.org/10.3201/eid2206.151565

Use of Population Genetics to Assess the Ecology, Evolution, and Population Structure of Coccidioides, Arizona, USA

Technical Appendix 3

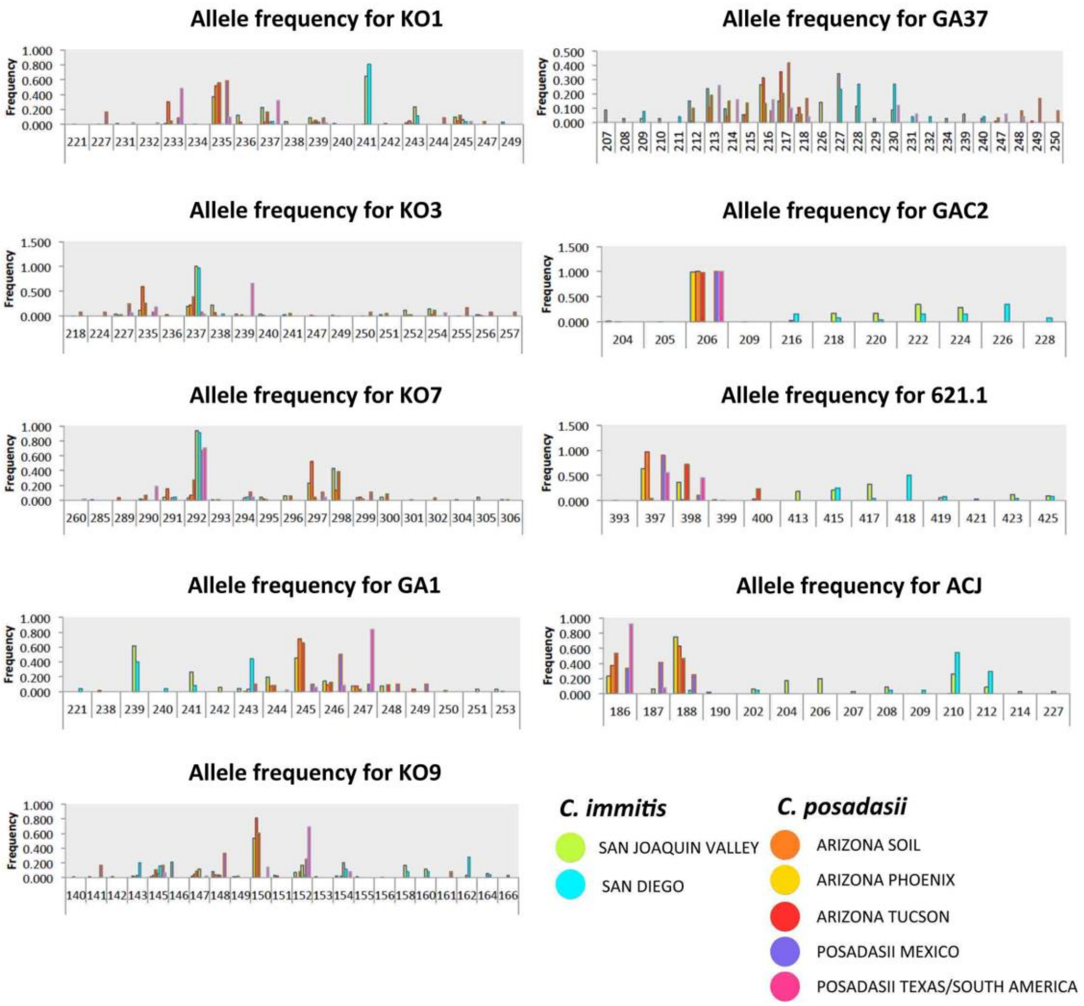

Technical Appendix 3 Figure 1. Microsatellite allele frequencies. Vertical axes are percentage of the population with that allelic size class. Horizontal axes are the size classes for each locus. Color coding is consistent with population schema detailed in Figure 1.

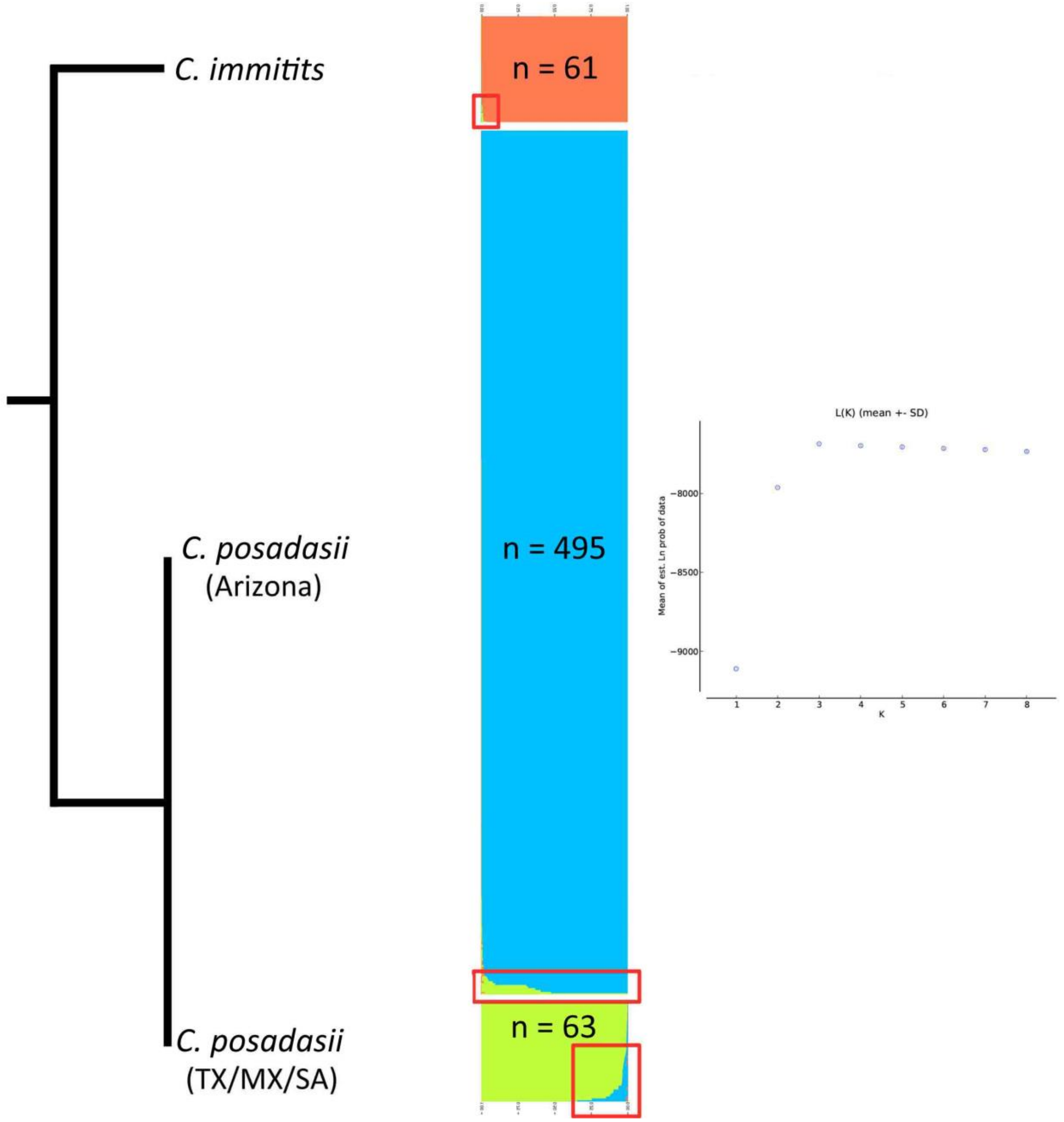

Technical Appendix 3 Figure 2. The population tree was generated displaying the three main populations of *Coccidioides*, number of individuals for each population as well the population distribution along the bar plots. Analysis of *Coccidioides* ssp. clustering using StructureHARVESTER and L(K) (mean +/– SD) shows optimal number of K clusters is 3 for the entire *Coccidioides ssp.* dataset.

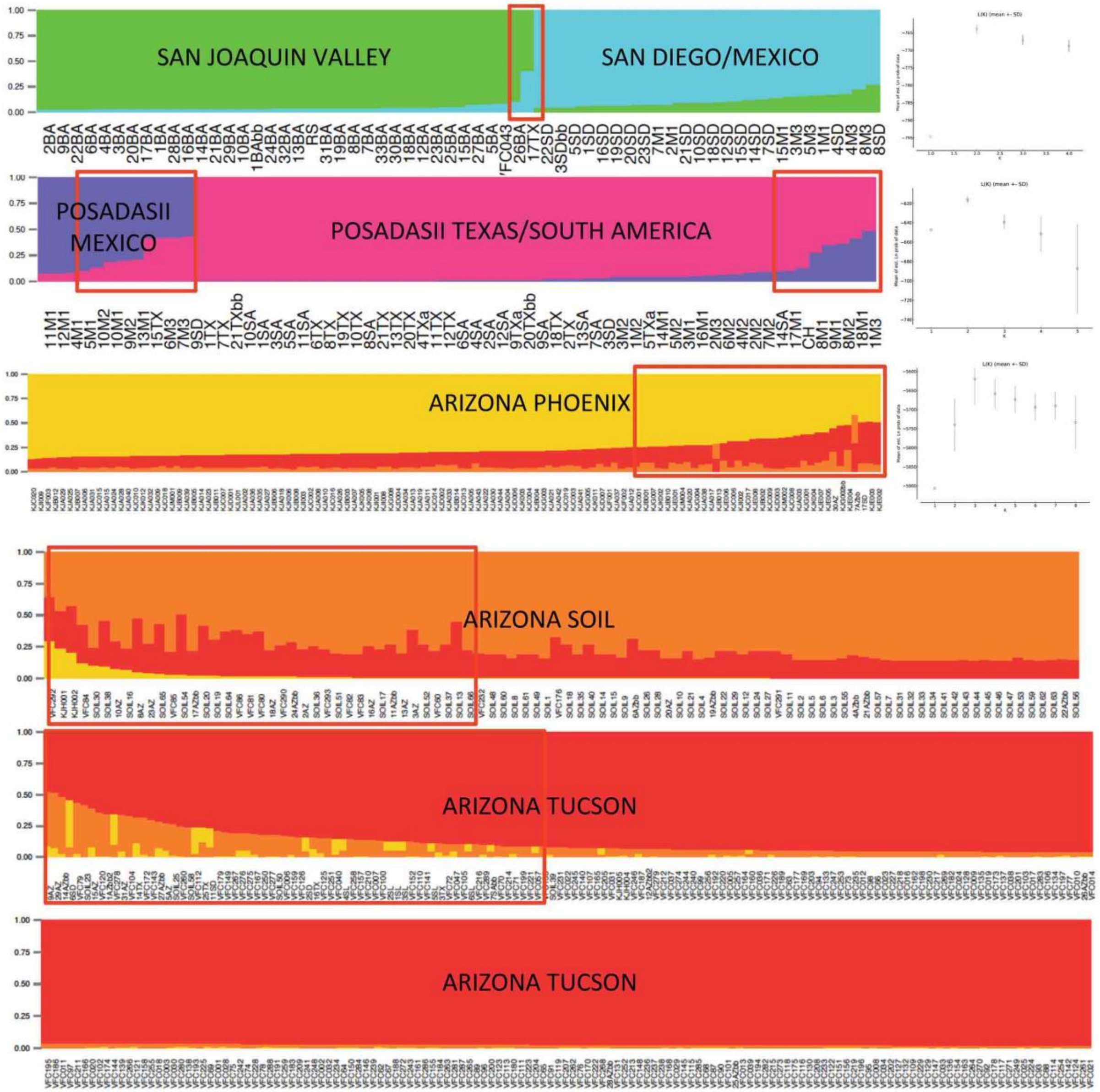

Technical Appendix 3 Figure 3. Individual bar plots for each of the seven population diagnosed in the current work (bottom of the plots). Shared plots show that limited gene flow were observed within *C. immitis* and *C. posadasii* sub-populations (boxed areas). Analysis of *Coccidioides* ssp. clustering using StructureHARVESTER and L(K) (mean +/– SD) shows optimal number of K clusters is 3 for *C. posadasii* Arizona, 2 for *C. posadasii* TX/MX/SA and 2 for *C. immitis*.
